# Study Protocol for the Evaluation of Morphologic and Imaging Remodeling of Atherosclerotic Plaque Following Intravascular Lithotripsy in Peripheral Artery Disease

**DOI:** 10.3390/jcm15083073

**Published:** 2026-04-17

**Authors:** Katerina Sidiropoulou, Athanasios Saratzis, Nikolaos Saratzis, Konstantinos Tigkiropoulos, Christos Karkos, Dimitrios Karamanos

**Affiliations:** 1 Division of Vascular Surgery, 1st Surgical Department, Faculty of Health Sciences, Aristotle University of Thessaloniki, Papageorgiou General Hospital, 56429 Thessaloniki, Greece; 2 NIHR Biomedical Research Centre, University of Leicester, Leicester LE3 9QP, UK; as875@leicester.ac.uk; 3 Division of Vascular Surgery, 5th Surgical Department, Faculty of Health Sciences, Aristotle University of Thessaloniki, Ippokratio General Hospital, 54642 Thessaloniki, Greece

**Keywords:** intravascular lithotripsy, study protocol, IVUS, peripheral artery disease

## Abstract

**Background:** Intravascular lithotripsy (IVL) has emerged as a novel vessel preparation device for patients with peripheral artery disease undergoing angioplasty. The IVL catheter includes an integrated balloon, which emits high pressure and transient sonic waves. The release of shockwaves results in cracking of intimal and medial calcium within the vessel wall improving lumen patency. **Objectives:** The aim of this prospective observational cohort study is to evaluate the morphological and imaging changes in atherosclerotic plaque in patients with PAD undergoing IVL as a vessel preparation technique, followed by angioplasty with drug-coated balloon (DCB) or stent placement if required. Secondary endpoint is to evaluate the efficacy of IVL in the perfusion of the lower extremities, by calculating the ankle–brachial index (ABI) and toe–brachial index (TBI) post-angioplasty, as well as adverse events within 30 days. **Methods:** Consecutive adult (≥18 years of age) patients with symptomatic femoropopliteal artery disease selected to undergo IVL will be included in the study. Computed tomography angiography (CTA) of the lower limbs will be performed pre- and postoperatively. Intraoperatively, an intravascular ultrasound (IVUS) will be used before and immediately post-angioplasty, for real-time evaluation of the morphological and quantitative changes in the atherosclerotic plaque. All participants will be clinically re-evaluated in 30 days postoperatively and a color Duplex ultrasound of the lower extremity arteries will be performed. The perfusion of the peripheral arteries will be assessed using ABI and TBI post-procedurally. **Outcomes:** The primary outcome is the quantitative assessment of changes in plaque morphology and volume within the index target lesion, based on pre- and post-procedural computed tomography angiography using TeraRecon™ (Durham, NC, USA) plaque analysis module, reflecting plaque modification and redistribution, in the context of IVL-based vessel preparation. Secondary outcomes include improvement of peripheral arterial perfusion and freedom from clinically driven target lesion revascularization (CD-TLR) and major adverse events.

## 1. Introduction

### 1.1. Intravascular Lithotripsy

Peripheral artery disease affects a significant number of patients worldwide, most of which require revascularization. Endovascular repair represents the first-line therapeutic approach for this condition and initially involved the use of plain balloon angioplasty, mainly for lesions of the femoropopliteal segment [1,2]. The balloon catheter is introduced and inflated, redistributing plaque volume from the center along the arterial wall, thereby reducing hemodynamic resistance and increasing both blood flow and arterial diameter. A major limitation of this method is the development of local vessel dissections and barotrauma due to the mechanical stress, which may lead to restenosis; an important drawback that does not favor plain balloon angioplasty as monotherapy [3,4]. For this reason, new devices were introduced in the armamentarium such as drug-coated balloons (DCB), cutting balloons and bare-metal and drug-eluting stents [5].

Drug-coated balloons release an antiproliferative agent into the vessel wall during balloon inflation and for as long as the balloon remains fully expanded. The drug possesses strong lipophilic properties, allowing improved and more homogeneous tissue uptake while remaining active within the vessel wall for a prolonged period. At present, two antiproliferative drugs have been approved for use in DCBs: paclitaxel and sirolimus. Although they differ in their molecular mechanisms of action, both inhibit smooth muscle cell proliferation and thereby reduce the risk of restenosis [6].

The use of stents in femoropopliteal angioplasty was initially reserved as a bailout option in cases of flow-limiting dissections following balloon angioplasty. Over time, stenting has become a primary treatment strategy in many patients with long lesions and occlusions of the superficial femoral artery, supported by growing clinical evidence showing better outcomes compared to plain balloon angioplasty. In most cases, self-expanding stents are preferred over balloon-expandable stents, as balloon-expandable platforms are more prone to recoil when exposed to significant mechanical stress [7].

Furthermore, atherectomy has been proposed for the treatment of heavily calcified lesions, which are often associated with higher rates of disease recurrence and in-stent restenosis. However, this technique may lead not only to removal of calcified plaque but also to damage of the healthy intimal layer of the vessel wall [8,9].

Intravascular lithotripsy is a relatively new technique derived from extracorporeal shockwave lithotripsy, which has long been used for the treatment of nephrolithiasis and ureterolithiasis, and is now increasingly applied in interventional cardiology. This technique employs an angioplasty balloon with integrated emitters that generate intermittent acoustic shockwaves. For the procedure to be performed in the femoropopliteal segment, lesion crossing and intraluminal advancing of a 0.014″ guidewire is required. The intravascular lithotripsy balloon catheter is then advanced across the atherosclerotic lesion and inflated to approximately 4 atm in order to achieve adequate apposition with the vessel wall and effective energy delivery. The emitters generate an electrical discharge that vaporizes the fluid inside the balloon, leading to the formation of a rapidly expanding and collapsing bubble, which in turn produces acoustic pressure waves. These waves propagate through the vessel wall and selectively fracture calcified plaque at the intimal and medial levels, while largely preserving surrounding soft tissue. Following plaque modification, the balloon is further expanded to 6 atm to achieve maximal luminal gain [10,11]. As IVL does not result in plaque removal, adjunctive balloon angioplasty or drug-coated balloon treatment is typically required. In addition, the technique is associated with a low risk of distal embolization, while complications such as vessel perforation or dissection are uncommon [12].

Each IVL M5+ balloon can deliver up to 300 pulses. It is generally recommended to perform three treatment cycles per lesion, with each cycle consisting of 30 pulses. When selecting the appropriate balloon diameter, an oversizing of approximately 10% compared to the diameter of a normal reference vessel segment is usually advised.

According to published studies [11,13,14,15,16], IVL has been shown to be a safe and effective vessel preparation technique prior to subsequent angioplasty with a drug-coated balloon or stent implantation. This is particularly relevant in patients with extensive atherosclerotic disease, who are at increased risk of recurrence and restenosis.

### 1.2. Intravascular Ultrasound (IVUS)

Intravascular ultrasound (IVUS) represents a particularly valuable intraoperative imaging modality during endovascular lithotripsy procedures. IVUS provides a 360° cross-sectional visualization of the vascular lumen from within the vessel itself and contributes to detailed assessment of atherosclerotic plaque morphology, quantification of atherosclerotic burden, and appropriate sizing of the balloon or stent to be used during angioplasty based on vessel diameter. In addition, it allows immediate evaluation of the angioplasty outcome, identification of plaque fractures, and detection of intraprocedural complications, such as vessel dissection or residual stenosis. Although IVUS is not routinely required after IVL, it may offer additional insights, including more precise evaluation of lumen gain, visualization of calcium modification, and detection of dissections not evident on angiography.

IVUS employs a piezoelectric transducer located at the tip of the catheter, which generates acoustic waves following electrical excitation. The propagation of these waves through different tissues produces images based on the acoustic properties of each tissue type. Typically, transducers operating at frequencies of 20–40 MHz are used [17,18,19,20].

### 1.3. Study Justification

Intravascular lithotripsy (IVL) is a novel and highly innovative endovascular technique that is gradually gaining ground in the management of patients with peripheral arterial disease. Current evidence regarding its efficacy and post-procedural complications remains relatively limited and is based on studies with small patient cohorts. Examples include the DISRUPT PAD I [11], DISRUPT PAD II [13], and the more recent DISRUPT PAD III [14] trials.

In the international literature, only one small independent study by Lopez-Pena et al. [21] has evaluated the effect of IVL on atherosclerotic plaque in patients exclusively with chronic limb-threatening ischemia, using CT angiography as the sole imaging modality across lesions involving the entire lower limb arterial tree.

The present study focuses on the effect of IVL specifically in lesions across the femoropopliteal segment—the most common site of steno-occlusive PAD, including patients with intermittent claudication, who represent the majority of cases with PAD. Moreover, the study incorporates intravascular ultrasound (IVUS) for detailed plaque characterization, while also assessing peripheral limb perfusion, and includes a pragmatic all-comers approach, involving all patients with femoropopliteal disease selected to have IVL in our unit. Finally, this is the first study to include detailed IVUS analysis of the plaque(s) treated and a standardized pre-/postoperative computed tomographic lesion assessment protocol, alongside ultrasonography (dynamic artery assessment).

### 1.4. Study Rationale

The present study aims to evaluate the effect of IVL on atherosclerotic plaque, specifically whether it induces a statistically significant remodeling and reduction in plaque burden as assessed by CT angiography (CTA).

Given that IVL does not result in true plaque debulking but rather induces calcium fracture and plaque modification, any observed reduction in plaque volume is interpreted as an apparent reduction reflecting plaque redistribution and early vessel remodeling rather than true plaque removal, particularly in the context of adjunctive endovascular therapies. In addition, real-time assessment using intravascular ultrasound (IVUS) will allow detailed evaluation of the immediate plaque response to IVL and the detection of any peri-procedural complications.

This approach is expected to provide further evidence and validation regarding the effectiveness of IVL in the treatment of femoropopliteal atherosclerotic disease, while also laying the groundwork for future studies comparing the impact of alternative endovascular techniques on plaque morphology and potential arterial compliance.

## 2. Methods

### 2.1. Study Design

This is a prospective, observational, single-center, single-arm study conducted in a tertiary institution, i.e., Papageorgiou Hospital of Thessaloniki. Patients with PAD who fulfill the inclusion criteria and none of the exclusion criteria will be consecutively enrolled. Written informed consent will be obtained from all patients. Patient demographics, medical history, and preoperative data will be recorded.

All patients must undergo preoperative CT angiography (CTA) of the lower extremities to confirm the diagnosis of PAD and the presence of calcified lesions. Intraoperatively, after successful intraluminal crossing of the target lesion, digital subtraction angiography (DSA) and intravascular ultrasound (IVUS) will be performed before and after IVL. Intravascular lithotripsy will be performed using the Shockwave™ Peripheral IVL System (Shockwave Medical, Inc., Santa Clara, CA, USA). Following successful lesion crossing, the IVL catheter will be advanced to the target lesion. Balloon sizing will be based on IVUS-derived reference vessel lumen diameter, with an intended oversizing of approximately 10%. The balloon will initially be inflated to 4 atm to ensure adequate apposition to the vessel wall and effective energy delivery. Intravascular ultrasound imaging will be performed using a 0.018″ IVUS catheter (OptiCross™ HD, Boston Scientific Corporation, Marlborough, MA, USA), in order to obtain measurements of lumen and vessel diameter, assess plaque modification, and detect the presence of dissection. IVUS measurements will be performed at predefined proximal and distal reference segments, with consistent localization ensured using pullback acquisition and bookmarking.

Following IVL, adjunctive treatment with drug-coated balloon angioplasty or stenting will be performed when clinically indicated, reflecting routine clinical practice. All patients will undergo CTA of the lower extremities within 7 days postoperatively. Pre- and postoperative CTA images will be analyzed using the TeraRecon™ software (version 4.9.0.29-82, Durham, NC, USA). Where feasible, imaging analysis will be performed in a blinded fashion to minimize observational bias.

The ankle–brachial index (ABI) and toe–brachial index (TBI) will be calculated preoperatively and immediately after angioplasty. Details regarding intraoperative findings and perioperative complications will also be recorded.

**Figure 1:**
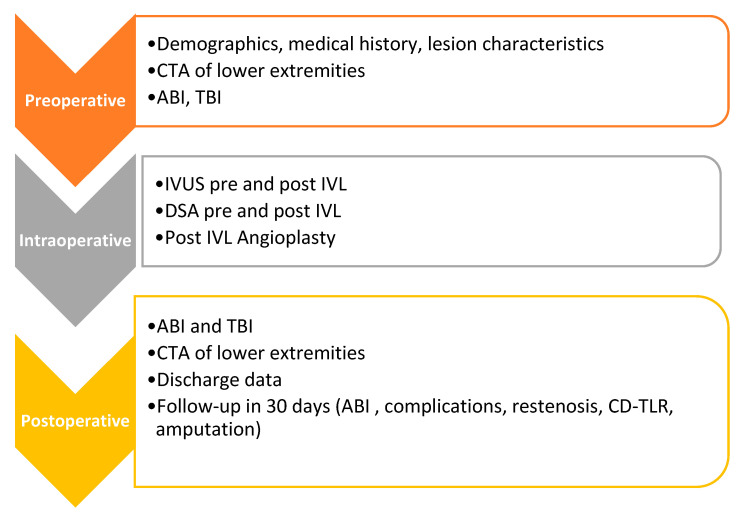
Study flow diagram.

All patients will receive dual antiplatelet therapy post-angioplasty. A 30-day follow-up visit is planned, during which complications, including restenosis, clinically driven target lesion revascularization (CD-TLR), ABI values, all-cause mortality, and major or minor amputation, will be documented. Study flow diagram is presented in Figure 1.

### 2.2. Sample Size

The sample size for this study was calculated to ensure adequate statistical power for detecting a significant change in atherosclerotic plaque volume. Using G*Power software (version 3.1.9.7; Heinrich Heine University Düsseldorf, Düsseldorf, Germany), a sample size calculation was performed to compare mean values before and after the intervention using a paired t-test.

The calculation was based on an effect size of Cohen’s d = 0.5 (medium effect size), which was conservatively selected to detect a mean difference equal to half the standard deviation and to ensure clinical relevance. This assumption was supported by previous studies on intravascular lithotripsy and plaque reduction in patients with peripheral arterial disease, which reported moderate-to-large effect sizes in similar interventions [10,21].

**Figure 2:**
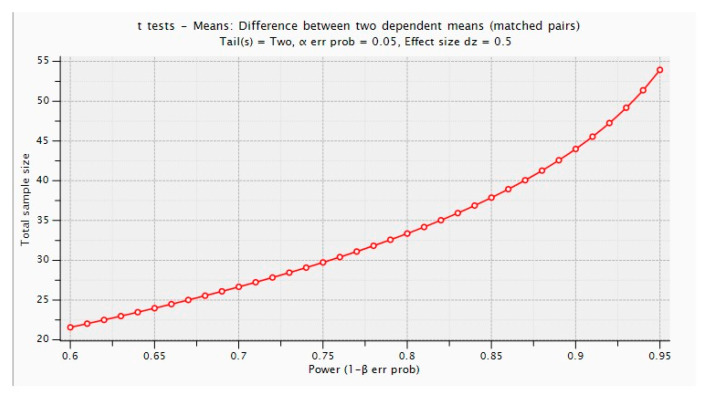
G*power output.

To achieve a statistical power of 80% with a two-sided type I error rate of α = 0.05, the required sample size was calculated as 34 patients. To account for potential dropouts, an additional 10% was included, resulting in a final target sample size of 38 patients. The G*Power output is provided for reference (Figure 2).

### 2.3. Inclusion/Exclusion Criteria

The study population will consist of adult patients with symptomatic peripheral arterial disease (PAD) of the lower extremities, who presented for scheduled endovascular angioplasty. All patients must have at least 2 patent tibial vessels run-off, to ensure a more homogeneous study population and to reduce the confounding effect of severe distal disease on perfusion and imaging assessment. (Table 1).

### 2.4. Inclusion Criteria

1. Adult male and female patients.
2. Symptomatic PAD of the lower extremities (intermittent claudication or critical limb ischemia), specifically with atherosclerotic stenoses or occlusions of the femoropopliteal axis, as documented by preoperative CT angiography.
3. At least 2 patent tibial vessels run off.

### 2.5. Exclusion Criteria

1. Asymptomatic patients.
2. Stenoses or occlusions of the target vessels exclusively of thrombotic/fibrotic etiology, as confirmed by preoperative CT angiography of the lower extremities.
3. Concomitant significant stenoses of the tibial arteries.
4. Patients presenting with acute limb ischemia or requiring urgent revascularization procedures.
5. Patients unwilling or unable to provide informed consent or to comply with study requirements.

### 2.6. Outcomes

The primary endpoint is the change in atherosclerotic plaque volume following intravascular lithotripsy (IVL), as assessed by pre- and postoperative CT angiography (CTA) using TeraRecon™ quantitative plaque analysis. This reflects plaque modification and redistribution, while acknowledging that CTA-based evaluation enables whole-lesion assessment but may be subject to imaging-related limitations.

### 2.7. Secondary Endpoints

1. The impact of IVL on lower limb perfusion, assessed by calculating the ankle–brachial index (ABI) and toe–brachial index (TBI).
2. Measurement of the increase in intraluminal diameter following IVL angioplasty at the site of maximum stenosis or occlusion, as determined by intravascular ultrasound (IVUS).
3. Detection of intraoperative complications (residual stenosis, vessel dissection), as documented by IVUS.
4. Target vessel patency at 30 days, assessed by clinical examination and Duplex ultrasonography.
5. Evaluation of postoperative vascular complications at 30 days, including major and minor lower limb amputations, reintervention for revascularization, major adverse cardiovascular events (MACE), and access site complications.
6. Presence of calcium fractures following IVL, as assessed by intravascular ultrasound (IVUS), with supportive evaluation by CTA plaque analysis when applicable.

### 2.8. Data Collection

Patient demographic and clinical characteristics will be recorded, including age, sex, body mass index (BMI), as well as comorbidities such as arterial hypertension, dyslipidemia, diabetes mellitus, chronic obstructive pulmonary disease, chronic kidney disease, coronary artery disease, atrial fibrillation, carotid artery disease, and previous ischemic stroke. Smoking status and the use of antiplatelet agents prior to angioplasty will also be documented.

Subsequently, the symptomatic limb will be identified, and the stage of peripheral arterial disease will be classified according to the Rutherford classification. Pre- and postoperative ABI and TBI values will be recorded.

Lesion-specific characteristics will then be documented, including lesion location (SFA or popliteal artery), type (stenosis or occlusion), morphology (eccentric or concentric), length, TASC classification, and PACSS score. Intraoperative data will also be collected, such as access vessel, type of anesthesia, use of pre-dilatation, size of the intravascular lithotripsy balloon, number of IVL balloons used, subsequent angioplasty with a drug-coated balloon (if performed), and stent placement following IVL in cases of residual stenosis.

The presence or absence of vessel dissection, as assessed by IVUS and digital subtraction angiography (DSA), will be recorded. Vessel lumen diameter at the site of greatest stenosis or occlusion will be measured pre- and post-procedure by IVUS.

Finally, lumen volume, as well as the morphology and volume of the atherosclerotic plaque (soft, fibrocalcific, or calcified), will be quantified using the TeraRecon™ software based on Hounsfield unit (HU) measurements.

For the primary endpoint, defined as the change in atherosclerotic plaque morphology and volume, lower limb CT angiography (CTA) will be performed preoperatively and postoperatively. The datasets will be analyzed using the TeraRecon™ software, which allows quantification of plaque volume and characterization of plaque morphology based on HU thresholds. Previous studies [22,23] have demonstrated that HU values between ࢤ100 and 100 correspond to soft plaque, 101–300 HU to fibrocalcific plaque, and 301–1000 HU to calcified plaque (Table 2).

Regarding the secondary endpoints, ABI and TBI will be measured. ABI will be calculated as the ratio of the highest systolic pressure of the ankle arteries to the highest systolic brachial pressure. TBI will be calculated as the ratio of the systolic pressure of the great toe to the highest systolic brachial pressure. These measurements will be performed using a sphygmomanometer, a Doppler device, and a plethysmograph, respectively.

Calcium fractures will be assessed on post-IVL IVUS imaging and recorded as present or absent. When feasible, CTA-based plaque analysis using TeraRecon™ software will be used to support imaging findings.

### 2.9. Statistical Analysis

Descriptive statistics will summarize the baseline demographic and clinical characteristics of the study population. Quantitative variables will be expressed as mean ± standard deviation (mean ± SD) with 95% confidence intervals when normally distributed, and as medians with interquartile ranges (25th–75th percentile) when non-normally distributed. Qualitative variables will be expressed as frequencies (n) and relative frequencies (%).

To ensure the appropriateness of the statistical analyses, variables will be categorized as quantitative or qualitative (dichotomous when relevant), and normality will be assessed using the Shapiro–Wilk test. The choice between parametric and non-parametric methods will be based on distribution characteristics.

For the primary endpoint, the change in atherosclerotic plaque volume before and after treatment will be analyzed using a paired t-test (if normally distributed) or the Wilcoxon signed-rank test (if non-normally distributed). In this context, changes in plaque volume will be interpreted as apparent changes reflecting plaque modification and redistribution.

Secondary endpoints, including changes in ankle–brachial index (ABI), toe–brachial index (TBI), and intravascular ultrasound (IVUS) measurements, will be analyzed using the paired t-test or the Wilcoxon signed-rank test, as appropriate.

Univariable linear regression analysis will be performed to explore potential predictors of apparent changes in plaque volume and perfusion indices. Multivariable analysis will be considered cautiously, given the sample size, and limited to a small number of clinically relevant variables to minimize the risk of overfitting, in order to further investigate the relationship between these changes and key lesion characteristics, including lesion length, TASC classification, and plaque morphology (e.g., eccentric versus concentric distribution).

Vessel patency at 30 days will be evaluated using Kaplan–Meier survival analysis. Complication rates will be compared using Fisher’s exact test or the chi-square (χ^2^) test, as appropriate.

The presence of calcium fractures will be reported as a categorical variable. Associations between calcium fractures and apparent changes in plaque volume will be explored using the independent samples t-test or the Mann–Whitney U test, as appropriate.

A subset of imaging measurements will be re-evaluated to assess inter- and intra-observer variability using appropriate statistical methods (e.g., intraclass correlation coefficient).

Given the relatively small sample size, this study is designed as a pilot, hypothesis-generating study, reflecting the limited availability of comparable imaging-based data in the current literature. Accordingly, all analyses will be interpreted cautiously to avoid overestimation of effect size.

For all hypothesis testing, *p*-values < 0.05 will be considered statistically significant. Data analysis will be performed using IBM SPSS Statistics for Windows, version 25 (IBM Corp., Armonk, NY, USA).

## 3. Limitations

This study has several limitations. First, it is a single-center study, which may limit the generalizability of the findings to other populations and clinical settings. Second, the relatively small sample size reflects the pilot nature of the study, which was designed to explore imaging-based plaque remodeling in a clinical setting where comparable data remain limited, and may limit the ability to detect smaller differences in plaque characteristics and clinical outcomes.

In addition, the observational design does not allow direct comparison with other vessel preparation techniques and may be subject to selection bias. Furthermore, the follow-up period is limited to 30 days, which may not fully reflect the long-term clinical and morphological effects of intravascular lithotripsy. The present study is focused on the assessment of early imaging-based changes rather than long-term vascular remodeling, as plaque characteristics may evolve over time under the influence of patient-related factors and medical therapy. Post-procedural CTA imaging findings reflect the combined effect of IVL and subsequent endovascular treatment (DCB angioplasty or stenting), which may confound the isolated assessment of IVL-induced changes. Finally, plaque characterization is primarily based on CTA-derived Hounsfield unit measurements, which may be influenced by technical and patient-related factors, including blooming artifacts in heavily calcified lesions, potentially affecting the accuracy of plaque volume estimation.

## 4. Conclusions

This prospective study will evaluate imaging-based plaque changes following IVL in femoropopliteal disease using quantitative CTA and IVUS assessment. The findings are expected to contribute to the understanding of plaque modification mechanisms and inform future endovascular strategies.

## Figures and Tables

**Table 1:** Inclusion and exclusion criteria.

Inclusion Criteria	Exclusion Criteria
1. Age > 18 years	1. Asymptomatic PAD
2. Symptomatic PAD (Intermittent claudication or CLTI)	2. Acute limb ischemia
3. Femoropopliteal atherosclerotic lesions	3. Non-atherosclerotic etiology
4. At least 2 patent tibial run-off vessels	4. Significant disease of the tibial vessels
	5. Inability to provide consent or comply with study protocol

**Table 2:** Hounsfield unit ranges for plaque components.

Plaque Morphology	Hounsfield Unit Ranges
Soft	−100 to 100
Fibrocalcific	101 to 300
Calcified	301 to 1000

## Data Availability

This manuscript represents a study protocol, and no datasets have been generated or analyzed at this stage. Data will be collected as the study progresses and will be reported in subsequent publications.

## Abbreviations

SFA = superficial femoral artery, CTA = computed tomography angiography, IVUS = intravascular ultrasound, IVL = intravascular lithotripsy, ABI = arterial-brachial index, TBI = toe–brachial index, CD-TLR = clinically driven target lesion revascularization, PACSS = Peripheral Arterial Calcium Scoring System, TASC = TransAtlantic Inter-Society Consensus.
